# News and Coming Events

**Published:** 1908-10-31

**Authors:** 


### News and Coming Events.

The Londonderry Cottage Hospital has been handed over by Lord Herbert Vane-Tempest to the Machynlleth Nursing Association.

An orchestral concert will be given at the Æolian Hall this afternoon (Saturday, October 31) in aid of the funds of the Hospital for Women, Soho Square.

At a meeting of the Senate of the University of London, held on October 21, the Vice-Chancellor (Sir William Collins, M.D., M.P.) being in the chair, the Rogers prize of £100 was divided equally between Dr. David Forsyth, M.D., M.R.C.P., and Mr. F. W. Twort, L.R.C.P., M.R.C.S. The essay of the former dealt with anatomy, physiology, and pathology of the thyroid and para-thyroid glands, and that of the latter with the fermentation of glucosides by bacteria of the typhoid-coli group. The degree of D.Sc. in physiology was conferred upon Dr. David Forsyth, of Guy's Hospital Medical School, for a thesis entitled "The Parathyroid Glands."

Mr. Leopold Rothschild presided last week at the New Ealing Hospital festival dinner in Victoria Hall, Ealing. The object of the gathering was to raise funds for the new hospital, which will cost about £20,000. At present £5,279 has been given or promised. During the evening the chairman announced further donations of over £900.

The Home Secretary has issued a draft of regulations which he proposes to make in pursuance of Section 79 of the Factory and Workshop Act, 1901, for certain branches of the metal-grinding industry in view of "the heavy mortality among grinders from phthisis, due to the dust generated," such mortality having been "a matter of grave concern to the employers and workmen and to the Home Office." The regulations are substantially in the form which, after careful consideration and discussion with the Home Office, has been unanimously recommended to Mr. Gladstone by the Cutlers' Company, the Sheffield Chamber of Commerce, and (as representing the workmen) the Sheffield Cutlery Council.

In support of the funds of the Poplar Hospital Mr. Abraham has started a series of Sunday Entertainments at the Queen's Palace of Varieties, Poplar.

The King has been pleased to give directions for the appointment of Dudly Cox Trott, Esq., M.B., to be a member of the Executive Council of the Bermudas or Somers Islands.

A charity concert in aid of the funds of St. George's Hospital and of the Central London Ophthalmic Hospital will be given by Madame Donalda at Queen's Hall on Friday, November 27, at three o'clock.

Mrs. Elizabeth Garrett Anderson, M.D., who was elected a Councillor last year, has been asked to accept the office of Mayor of Aldeburgh for the ensuing year. Her late husband, her father, and her brother were all, at various times, Mayors of Aldeburgh.

After providing for the payment of legacies, Mr. J. Briscoe, F.R.C.S., of Oxford, formerly surgeon to the Radcliffe Infirmary, Oxford, whose estate has been valued for probate at £79,294, left the residue of his property, amounting to about £67,000, to the infirmary.

The King has granted George Allardice MacDonald, Esq., M.B., C.M., his Majesty's Royal licence and authority to accept and wear the Insignia of a Knight of the Order of the Crown of Italy, conferred upon him by his Majesty the King of Italy, in recognition of valuable services rendered by him.

Dr. P. H. Pye-Smith, M.D., B.A., F.R.S., was re-appointed, on October 22, to represent the University of London on the General Medical Council, and Dr. E. A. Gates, M.D., was appointed to represent the University on the occasion of the Torricelli Tercentenary Celebration, held at Faenza.

In the calendar of the Royal College of Surgeons just published there appear 1,413 Felows on the roll of the College—an increase of 26 compared with the number last year. There are 16,928 members and 363 licentiates in midwifery. The licentiates in dental surgery number 2,109. Holders of the diploma in public health granted by the Royal Colleges of Physicians and Surgeons number 615. The income of the College from all sources amounted to £24,974 13s. 3d., of which the fees paid by candidates for diplomas produced £16,164 4s. 6d. The expenses of the year amounted to £21,181 0s. 9d., of which examiners' fees comprised £6,677 12s. From June 1, 1907, to June 1, 1908, 12,769 people visited the museum. During the collegiate year the library was open on 252 days, and was used by 10,244 readers.

A preliminary meeting has been held at Glasgow for the purpose of raising a suitable memorial to the late Dr. J. B. Russell, who served the city for many years with great distinction as Medical Officer of Health, and vacated that office to become medical member of the Local Government Board for Scotland. Sir William Bilsland, the Lord Provost, who was closely associated with Dr. Russell during a large part of his career, presided over the meeting, and spoke in the warmest terms of Dr. Russell and his life-work. When he joined the public health service of the city, the death-rate was 33 per 1,000, and there were 1,177 deaths in one year from typhus fever alone; during his last year of office the death-rate was 21.2, and there were only six deaths from typhus. It has been decided to place a bust of him in the City Art Galleries in Kelvingrove, and a replica in the City Chambers.

The Royal Sea Bathing Hospital at Margate has received from an old subscriber the final instalment of a donation of £2,000 for the endowment of the Hugh Taylor free bed.

The students of Edinburgh University on Saturday last elected Mr. George Wyndham, M.P., Lord Rector of the University. The three candidates were Mr. Wyndham (Unionist), Mr. Winston Churchill, M.P. (Liberal), and Professor William Osler, M.D. (Independent). The contest was exceedingly keen, and the greatest excitement prevailed. At half-past twelve o'clock Principal Sir William Turner declared the result of the poll as follows: Mr. Wyndham, 826; Mr. Churchill, 727; Dr. Osler, 614.

The Council of Ministers has sanctioned the introduction of a Bill in the Russian Duma amending the regulations for the sale of spirits. The consumption of spirits is to be restricted by increasing the capacity of the smallest measure that can be sold to half a gallon, and by reducing the number of places where spirits can be bought. The inhabitants of villages are further given the right to close existing public-houses, to prohibit the opening of new ones, and to limit the hours of sale by resolution of the local council. The Bill also provides for the prosecution in the criminal Courts of habitual drunkards, and imposes penalties for the illicit sale of spirits.

Lord Farquhar and Sir Richard Martin, treasurers of the Royal National Orthopædic Hospital, have received a further donation of £26 5s. towards the rebuilding fund of that institution from the Worshipful Company of Fishmongers. This brings the Company's grant up to £52 10s. A special appeal is being made for £25,000 to provide more adequate accommodation. A special *matinée* with this object is to be given by permission of Mr. George Alexander at the St. James's Theatre on December 3. Many of the most prominent actors and actresses in London will appear, and seats may now be booked at the hospital, 234 Great Portland Street, or at the St. James's Theatre.

The death occurred, on October 20, of Dr. Cuthbert Collingwood, M.A., and B.M. Oxon, F.L.S., M.R.C.P., of Lewisham, in his eighty-second year. Dr. Collingwood was greatly distinguished as a botanist. He was educated at King's College School, Christ Church, Oxford, Edinburgh University, and Guy's Hospital. In 1849 he graduated at Oxford, and in 1859 was admitted a member of the Royal College of Physicians. From 1858 to 1866 he was lecturer in botany at the Medical School, Liverpool, and in 1866 made a scientific voyage to the Far East to study marine zoology. The results of his investigations have been recorded in his "Rambles of a Naturalist on the Shores and Waters of the China Seas" and many articles in scientific journals.

The death occurred on October 25, at the age of seventy-four, of Dr. James Adam, resident physician and proprietor of the West Malling Place Private Asylum for the Insane. Dr. Adam was educated at Edinburgh University, and took his M.D. degree at St. Andrews in 1860. He had held the post of senior assistant medical officer and acting superintendent of the female department of the Middlesex County Asylum, Colney Hatch, and he was honorary local secretary and treasurer of the Royal Medical Benevolent College. He was formerly medical superintendent of the Metropolitan District Asylum, Caterham, and of the Crichton Royal Institution and Southern Counties Asylum, Dumfries.

